# Enabling Better Use of Person-Generated Health Data in Stroke Rehabilitation Systems: Systematic Development of Design Heuristics

**DOI:** 10.2196/17132

**Published:** 2020-07-28

**Authors:** Gerardo Luis Dimaguila, Kathleen Gray, Mark Merolli

**Affiliations:** 1 School of Computing and Information Systems University of Melbourne Melbourne Australia; 2 Centre for Digital Transformation of Health University of Melbourne Melbourne Australia

**Keywords:** person-generated health data, patient-reported outcome measures, heuristics, stroke rehabilitation, consumer health informatics, evidence-based practice, information technology

## Abstract

**Background:**

An established and well-known method for usability assessment of various human-computer interaction technologies is called heuristic evaluation (HE). HE has been adopted for evaluations in a wide variety of specialized contexts and with objectives that go beyond usability. A set of heuristics to evaluate how health information technologies (HITs) incorporate features that enable effective patient use of person-generated health data (PGHD) is needed in an era where there is a growing demand and variety of PGHD-enabled technologies in health care and where a number of remote patient-monitoring technologies do not yet enable patient use of PGHD. Such a set of heuristics would improve the likelihood of positive effects from patients’ use of PGHD and lower the risk of negative effects.

**Objective:**

This study aims to describe the development of a set of heuristics for the design and evaluation of how well remote patient therapeutic technologies enable patients to use PGHD (PGHD enablement). We used the case of Kinect-based stroke rehabilitation systems (K-SRS) in this study.

**Methods:**

The development of a set of heuristics to enable better use of PGHD was primarily guided by the R3C methodology. Closer inspection of the methodology reveals that neither its development nor its application to a case study were described in detail. Thus, where relevant, each step was grounded through best practice activities in the literature and by using Nielsen’s heuristics as a basis for determining the new set of heuristics. As such, this study builds on the R3C methodology, and the implementation of a mixed process is intended to result in a robust and credible set of heuristics.

**Results:**

A total of 8 new heuristics for PGHD enablement in K-SRS were created. A systematic and detailed process was applied in each step of heuristic development, which bridged the gaps described earlier. It is hoped that this would aid future developers of specialized heuristics, who could apply the detailed process of heuristic development for other domains of technology, and additionally for the case of PGHD enablement for other health conditions. The R3C methodology was also augmented through the use of qualitative studies with target users and domain experts, and it is intended to result in a robust and credible set of heuristics, before validation and refinement.

**Conclusions:**

This study is the first to develop a new set of specialized heuristics to evaluate how HITs incorporate features that enable effective patient use of PGHD, with K-SRS as a key case study. In addition, it is the first to describe how the identification of initial HIT features and concepts to enable PGHD could lead to the development of a specialized set of heuristics.

## Introduction

### Varying Effects of Person-Generated Health Data

Person-generated health data (PGHD) are produced from technologies that allow people to access and utilize health data that they themselves generate outside of a health clinic setting, and to share these data with health care providers and others, typically via the internet. These technologies are designed to generate data about a range of health conditions and in pursuit of a range of health outcomes for remote patient monitoring [1,2]. Example technologies include home-based, web-based, mobile, and wearable apps, which cater to a range of health conditions such as irritable bowel syndrome [3], diabetes [4,5], and bipolar disorder [6]; text reminders for asthma management [7]; and social networking sites for the mental well-being of cancer patients [8].

It is possible for PGHD utilization to have positive, negative, or nil effects on patients who use these technologies. PGHD use has been reported to provide patients with a range of benefits, such as increasing interest in their own health care processes [5,9,10], allowing them to monitor and manage their own health status [11], and motivating them to undergo positive behavior change [1,12]. However, PGHD utilization can also cause patients to feel frustrated and discouraged [1], and some patients may feel excluded from the benefits of PGHD [11]. This highlights the need to consider patients’ perspective in the design and development of health technologies [13], particularly those that generate PGHD [14].

### Measuring PGHD Effects

The varying effects of PGHD use on patients necessitates the development of a patient-reported outcome measure (PROM) of utilizing PGHD, or PROM-PGHD [15], to provide a standardized way of measuring PGHD outcomes and build empirical evidence about PGHD [16]. PROMs are used to assess the health status or experience of health services and interventions by patients themselves [17-19] and have been shown to improve the precision of evaluating health information technologies (HITs) [20]. Similarly, PROMs-PGHD would measure patients’ health outcomes or status as a result of accessing and using PGHD. Moreover, they could complement other clinical health outcome indicators, similar to how PROMs are used alongside other biometric measurements of patient health [21].

The authors previously developed a PROM-PGHD that entailed establishing health outcomes that should be measured for a specific technology category and a health condition [15,22]. The process identified some features that a domain or type of PGHD-enabled technology, that is, stroke rehabilitation systems, should have to evaluate their effectiveness in producing positive effects on patients who use PGHD to self-monitor or manage a specific health condition. For example, simulated stroke rehabilitation technologies should provide patients with real time information on the remaining exercise repetitions they need to do for them to self-manage their limited energy [1].

### Evaluation of Technologies Using Heuristics

Using a list of features based on predetermined criteria to assess technology categories is called heuristic evaluation (HE). First introduced by Nielsen and Molich [23], HE is an established and well-known method for the usability assessment of various human-computer interaction technologies [24]. Using a set of guidelines, called heuristics, expert evaluators are able to quickly and efficiently identify issues that could affect the usability of technology artifacts [25]. In practice, it could also be conducted during the design process to identify potential problems before launch [24,25].

HE has since been adopted for evaluations in a wide variety of specialized contexts and with objectives that go beyond usability. This has resulted in specialized heuristics, which could be used to direct evaluators to focus on assessing technologies according to specific concepts of interest [24]. Nielsen’s original set of heuristics has been adopted or augmented to assess the quality of novice programing systems [26], identify common mistakes caused by novice and experienced users of a web-based health record system for nurses [27], and assess the safety of a HIT [28]. Such specialized heuristic evaluations have identified problems beyond the original scope of the original heuristics [29] and could therefore increase the effectiveness of evaluations in varied contexts [30] and improve the quality of insights gained [26].

### Heuristics for PGHD Enablement

Developing a set of heuristics for use in the formative stages of development of patient and consumer health technologies could ensure more deliberate PGHD-enabling designs. This would improve the likelihood of positive effects from patients’ use of PGHD and lower the risk of negative effects. Such heuristics would also offer health care providers a standard way to evaluate technologies as part of selecting and implementing PGHD programs with their patients [11]. HE would complement other in-depth PGHD evaluation methods [2,15] to explore PGHD outcomes [14,16], integration with clinical tools [2], and assessment of quality management [31]. A set of heuristics to evaluate how HITs incorporate features that enable effective patient use of PGHD is needed in an era where there is a growing demand and variety of PGHD-enabled technologies in health care and where a number of remote patient-monitoring technologies do not yet enable patient use of their PGHD. Furthermore, research into the outcomes and benefits of PGHD has not kept up [2,11,14]. Specifically, methods are only emerging for systematic measurement of patient-reported health outcomes from accessing and utilizing PGHD [11,15]. In contrast to the already strong standardization of PROMs in many aspects of health care [21], PGHD outcome evaluation methods are idiosyncratic and reported research is fragmented [11].

### Objective

In this study, we describe the development of a set of heuristics for the design and evaluation of how well remote patient therapeutic technologies enable patients to use PGHD (PGHD enablement). We used the case of Kinect-based stroke rehabilitation systems (K-SRS) to illustrate how the development of PROM-PGHD has led to the creation of these heuristics.

## Methods

### Case Study

An important use case for evaluating PGHD effects is in simulated rehabilitation technologies for stroke, in particular those using body-tracking technology Kinect (Microsoft) [15]. Key considerations within the context of stroke rehabilitation include the inherent complexity of care necessary to improve health outcomes [32,33], the requirement for stroke survivors to frequently conduct repetitive movement exercises [34,35], and the difficulty of accessing therapy accompanied by the high cost of care over an extended period [36].

K-SRS offer a more convenient and cost-effective option [14]. They allow stroke survivors to undertake prescribed movement exercises simulating activities of daily living (ADL) [37-40]. As survivors use these systems, they produce PGHD in the form of data indicating their therapeutic progress. These data, generally designed to be used by clinicians, have the potential to be used by stroke survivors themselves for health self-monitoring [14,38,39].

### Methodology to Develop Specialized Heuristics

A number of heuristic development processes have been employed in the literature. This includes literature reviews, analysis of usability problems, mixed processes, use of guidelines, interviews, and analysis of theories related to a specific domain [41]. Of these, only one methodology known as the R3C [42] has been used extensively (14 times) [41,43,44]. The authors have since improved it [45]. Another widespread approach is to point out the limitations of existing heuristics, in particular [23], and build on these by exploring other aspects that need to be considered for the target domain. The original set of usability heuristics did not detail the steps taken to develop them [23,46]. Subsequently reported processes for developing heuristics for specific application domains have varied.

### Procedure to Develop Heuristics for PGHD Enablement

The development of a set of heuristics for PGHD enablement was primarily guided by the R3C methodology most utilized and applied in the literature [45]. However, a closer inspection of the methodology reveals that neither its development [44] nor its application to a case study [47] were described in explicit detail, leaving room for flexibility and interpretation for future implementations. There are also overlaps between its steps and the other heuristic development processes described earlier [41]. For instance, step 1 explores and describes the target technology domain, followed by step 2, where the meaning of usability is reexamined within the context of the target technology domain. Thus, a literature review may be conducted for these steps [47]. Moreover, although those steps do not specify conducting qualitative activities such as interviews and focus groups, their goals may indeed be augmented by such activities.

Thus, the development of a specialized set of heuristics for K-SRS technologies was guided by the R3C [45]. Where relevant, each step was grounded through the best practice activities in the literature, that is, analysis of the context of use through a literature review, interviews, and focus groups, and by using the original heuristics [23] as a basis for determining the new set of heuristics. As such, this study builds on the R3C methodology, and the implementation of a mixed process is intended to result in a robust and credible set of heuristics. Moreover, the detailed implementation of each step is intended to aid future developers of specialized heuristics for PGHD enablement within various contexts of health conditions and technology domains.

The steps of R3C are described in Textbox 1. The objective of this study was to develop an initial set of heuristics for PGHD enablement; thus, only steps 1 to 4 are implemented in this study. Evaluation of the heuristic set (step 5) and further refinement (step 6) is an area for future research.

**Methodology to develop a specialized set of heuristics.**

Steps of the R3C methodology to develop a specialized set of heuristics [44,45,47]

- Step 1: Exploratory stage
  Examine literature related to the main topics or technology domain of the research.
- Step 2: Descriptive stage
  Highlight key concepts from step 1. Connect the information, and assign weights.
- Step 3: Correlational stage
  Identify the characteristics that the heuristics should have. Original heuristics [23] may be used as a basis. Clarify the need to consider the target technology domain.
- Step 4: Specification stage
  Formally specify the set of proposed specialized heuristics, using a standard template. Prioritize heuristics, and report any missing elements that need to be added.
- Step 5: Validation or experimental stage
  The new set of heuristics is tested on the target domain, and usability problems or issues found are compared with those that would be found using traditional heuristics.
- Step 6: Refinement stage
  The new set of heuristics is refined or improved following analysis of the results of step 5. However, this occurs iteratively, that is, if no more changes are recommended after step 5 then the development ends there.

## Results

### Step 1: Exploratory Stage

A literature review was conducted as part of the PROM-PGHD development process to understand how K-SRS have been designed to enable PGHD and what benefits, if any, were recorded [14] using them. Similar to how this step was applied for this case [47], the review also analyzed features of this domain, such as the effectiveness of K-SRS. Moreover, the types of PGHD generated by K-SRS were reviewed as well as how people were able to access those PGHD, the intended users and uses of PGHD, and any effects on patients from using PGHD [14].

The literature review described the context under which PGHD is produced and used, relevant for step 4 when the heuristics are specified. As part of this step, factors that may influence the outcomes resulting from accessing and using PGHD were analyzed. The review has shown that PGHD was given to patients to guide them, for example, change movement behavior or actions to perform correct exercise movements. PGHD were also provided to help them achieve their short- and long-term goals [14]. However, although people were generally provided with some form of PGHD as feedback, they did not have access to all their PGHD, which clinicians have access to. Other uses of PGHD include clinicians analyzing them to tailor rehabilitation programs for patients; researchers analyzing the effects on patients when they use a K-SRS; and to assess a variety of K-SRS, for example, how effective and reliable they are, and even compare them with other domains of technologies [14]. The review also identified one study that reported that a patient’s daily access to her PGHD caused her to remember them over time and be motivated to improve [48].

The review also highlighted the lack of patient-centered design in the development of K-SRS, given that the focus of providing data back to patients was for prescribing tasks and not to allow patients to access and make sense of their PGHD themselves. This is a missed opportunity to encourage patients to be more involved in their own health care [14], as patients who have direct access to their PGHD may become more engaged and thus improve their health outcomes [5,9,10,49]. Indeed, the lack of patient participation in the design of such PGHD systems may undermine patients’ rehabilitation experience [50]. This is indicative of the need for developers of technology-based rehabilitation systems to consider PGHD in their design, implementation, and evaluation and allow patients to access them [14].

### Step 2: Descriptive Stage

This step is described as having 3 tasks: highlighting, connecting, and assigning weights to the concepts found in step 1. Step 2 is applied for this study as follows. First, concepts found through the literature review that could enable the effective patient use of PGHD were identified. Second, the concepts were categorized based on their inherent meaning according to 5 different reported effects of PGHD use thematically derived from the literature. Articles from a significant journal special issue on PGHD were inductively analyzed as an efficient and targeted way to identify reported effects of PGHD utilization from a range of HITs for different health conditions [15]. This process identified 5 different effects that may result from patients’ use of their PGHD. PGHD may influence health-related behavioral or attitude changes, influence patients’ management of their own care owing to changes in how they feel about their health status, influence interest in their health care processes, facilitate their personal care goals, and influence their relationship with care providers. Concepts that did not match any of these effects were assigned a different category.

Table 1 shows the categorized concepts highlighted from the literature review. More example quotes are shown in Multimedia Appendix 1. The *Weight* column is discussed in the following paragraphs.

In the third activity of this stage, the concepts were assigned weights through qualitative studies. As part of the PROM-PGHD development process, the input of target users (stroke survivors) and experts (physiotherapists) was elicited through focus groups and interviews [51]. On the basis of the health and technology case under consideration, stroke survivors with experience interacting with a leading K-SRS called Jintronix [14,52] were asked to respond to open-ended questions around the effects resulting from their access to and utilization of PGHD. Meanwhile, the clinicians were asked how they thought that access and utilization of PGHD would affect the stroke survivors they were working with. Participant recruitment was conducted in Australia at 3 different sites, with ethics approval granted by the Human Research Ethics Committees of Deakin University (2017-087), Austin Health (HREC/17/Austin/492), and the University of Melbourne (1852259.1). A total of 10 stroke survivors (7 females and 3 males) were recruited through Deakin University; 5 clinicians (4 females and 1 male) and 6 stroke survivors (1 female and 5 males) were recruited through the Austin Health hospital; and 1 stroke survivor (female) was recruited through Headway ABI in Queensland, Australia.

The qualitative studies enabled the authors to receive direct feedback from PGHD users to assign weights to the concepts found from step 1. In addition, the studies also bridged any relevant gaps between the concepts described in the literature, those perceived by clinicians, and those reported as important by stroke survivors themselves [1]. New PGHD outcomes were documented that were not previously identified through the review and analysis of the literature described earlier [14], leading to the identification of new features that should be incorporated in technologies to enable effective patient use of PGHD.

Concepts identified through the literature review but not described by either stroke survivors or clinicians were assigned a weight of 0, concepts described by clinicians only were assigned a weight of 1, concepts described by stroke survivors only were assigned a weight of 2, and concepts described by both stroke survivors and clinicians were assigned a weight of 3. Table 1 shows the key concepts identified through the literature review assigned with weights and example quotes describing the concept, if any. Table 2 shows new concepts identified through the qualitative studies, also assigned with weights and listing example quotes.

The categories were initially applied by GD and then independently reviewed by the coauthors. Any disagreements in the categories applied were discussed in a meeting until agreement was reached for each concept. More example quotes are shown in Multimedia Appendix 1.

**Table 1:** Weighted key concepts identified from step 1: exploratory stage.

Categories	Key concepts	Weight
Stroke survivor health-related behaviors	PGHD^a^ can guide stroke survivors to make appropriate movement behavior or action changes to perform an exercise correctly	2—stroke survivors: “I automatically adjust what I was doing, if I couldn't burst every balloon I, I had to adjust… To work out what I was doing wrong” (FG1_STC2-3)
Stroke survivor personal care goals	PGHD can help stroke survivors achieve their short- and long-term goals	0—Not described
Functional effectiveness of therapy	PGHD can help inform clinicians, to analyze the functional effects of simulated rehabilitation therapy, and tailor programs for stroke survivors	0—Not described
Evaluation of the PGHD-enabled K-SRS^b^	PGHD can be used to assess the effectiveness and reliability of a K-SRS, compared with other types of simulated rehabilitation technologies	0—Not described
Stroke survivor interest in care processes	PGHD may be remembered by stroke survivors over time and can provide them with more motivation to improve their therapy performance PGHD needs to be accessible to the stroke survivors who produce them to allow them to be more involved in their own health care	3—stroke survivors and clinicians: PGHD may provide “an extra percentage of motivation” (FG1_STC2-1) and an “incentive to do better” (FG1_STC2-3) Clinician: “something to keep striving” (FG3_AHC1_3) 3—stroke survivors and clinicians: “it helped me understand my rehabilitation progress” (INT1_HWC1) “quite often you learn more when you've done something wrong” (FG2_AHP_2) Clinician: “[survivors] would understand better after a second session where they could compare their results...it's good for them to have a comparison, to see how they've changed from one session to another” (FG3_AHC1_5)

^a^PGHD: person-generated health data.

^b^K-SRS: Kinect-based stroke rehabilitation systems.

**Table 2:** Weighted new concepts identified from qualitative studies.

Categories	Key concepts	Weight
Stroke survivor health-related behaviors	PGHD^a^ can encourage stroke survivors to do more exercises related to their therapy PGHD can discourage stroke survivors from doing more exercises if it is negative or low	3—stroke survivors and clinicians: “if I can see the improvement I'm making then it would...encourage me to maybe have more of those sessions” (INT1_STC3) Clinician: “that might stimulate them to be more compliant” (FG3_AHC1_5) 3—stroke survivors and clinicians: “probably lose faith in the system” (INT2_AHP) [unless there is] “some explanation [...] that you need to consider looking” (INT2_AHP) Clinician: “They might get over it, or might not be willing to participate” (FG3_AHC1_3)
Stroke survivor personal care goals	PGHD can demotivate stroke survivors if it is negative	2—stroke survivors: “that can be a positive motivator, but can also (be) a negative one” (INT2_STC1) “I'm thinking well, are normal people at a hundred percent? And I'm only at 60?” (FG1_AHP_2)
Feelings about health status	PGHD can cause positive or negative emotions, correlated with whether their PGHD is positive or negative PGHD can make stroke survivors feel confused about their health progress PGHD can make stroke survivors feel more self-aware about their health care	3—stroke survivors and clinicians: “going backwards, that would be a little bit depressing” (INT1_AHP) seeing “yourself gradually making improvements, it just makes you feel so much better. Okay, I'm achieving something” (INT1_AHP) Clinician: “a score can motivate you or please you” (FG3_AHC1_5) 3—stroke survivors and clinicians: “the percentages to me is more difficult to understand [...] are we trying to be a hundred percent at these things?” (FG1_AHP_2) Clinician: “scope to put in something about, I didn't really understand” (FG3_AHC1_4) 2—stroke survivors: “to measure your improvement...or measure your, deterioration...You could see it in cold hard figures.” (FG2_AHP_2)
Stroke survivor interest in care processes	PGHD can interest stroke survivors in how their exercises are contributing to their activities of daily living PGHD can help stroke survivors to self-manage their energy while undergoing therapy PGHD can affect stroke survivors’ perception about their therapy	1—clinicians: “any relevance to a functional activity. You know like washing the dishes, or hanging the washing out or...climbing a flight of stairs” (FG3_AHC1_4) 2—stroke survivors: “It was certainly something that I watched, to see where I was at. 'Cause you need to think about this, we have some sort of a budget of energy that you have to manage yourself, and you can't afford to get to empty” (FG1_STC2-3) 3—stroke survivors and clinicians: Mismatch between PGHD and feeling of performance: “you probably think oh, the system's not doing its job” (INT2_AHP) “nothing going on in the background about anyone judging me...that there isn't anything that's being kept from me” (FG1_AHP_2) Clinician: if you just did the computer thing and they...just stopped without data they'd be like, why do I do it?” (FG3_AHC1_2)
Stroke survivor relationship with care provider(s)	PGHD can prompt stroke survivors to contact their therapists about their therapy performance PGHD can make stroke survivors be more conscious of the exercises prescribed by their clinicians	3—stroke survivors and clinicians: “If they were always bad then I would need more assistance and even if they [were] good, they [are] not perfect, right so I would want to have more, more assistance to improve” (INT2_STC3) Clinician: how can I get a better score...why did I...not do very well” (FG3_AHC1_4) 1—clinicians: “maybe if they're not being compliant, they might get a phone call from the therapist” (FG3_AHC1_5)
Relationship with family and carers	PGHD can assist stroke survivors in communicating their rehabilitation progress with their loved ones	3—stroke survivors and clinicians: “you could show them something, it's easier for them to visualize” (INT1_AHP) Clinician: “share it with family...it's that...bragging power as well, perhaps” (FG3_AHC1_4)

^a^PGHD: person-generated health data.

### Step 3: Correlational Stage

In this stage, the characteristics that the new set of heuristics should have are defined, which are later specified in step 4.

The key concepts identified through the first 2 steps are used as the basis to define the characteristics. As opposed to concepts, characteristics read as features that K-SRS should have to enable stroke survivors to use PGHD. These characteristics are also matched with the original heuristics [23] to identify any similarities. Some of the concepts were defined with similar characteristics and, therefore, combined. To further increase the credibility of the resulting heuristics, any characteristic matching an original heuristic is reworded using the original heuristic as a guide. Moreover, concepts with a weight of 0, that is, not described by stroke survivors or clinicians, were not defined as a characteristic and correspondingly not specified as a heuristic.

The characteristics were initially defined by GD and then independently reviewed by the coauthors. Any disagreements were discussed in a meeting until agreement was reached for each characteristic.

Table 3 lists the characteristics defined from the concepts identified previously that match an original heuristic [23], and as we have reworded them. Textbox 2 lists the characteristics that did not have a matching original heuristic [23]. The numbering continues from Table 3 through to Textbox 2, to indicate which characteristics were combined in the next step. To see which concepts were defined as which characteristics, please see Multimedia Appendix 1.

**Table 3:** Characteristics defined from the key concepts identified from steps 1 and 2—that match an original heuristic.

Number	Characteristics	Reworded using the original heuristic as a guide
1	PGHD^a^-enabled systems should assist users in performing more exercises or actions, in a correct way. Matched with original heuristic 9.	Help stroke survivors in performing more exercises or actions and to recognize, understand, and recover from errors they make. Guidance or error messages should be expressed in plain language (no codes), precisely indicate the problem, and constructively suggest a solution.
2	PGHD-enabled systems should provide PGHD to stroke survivors for increased understanding about their rehabilitation or therapy process. Matched with original heuristic 1.	PGHD-enabled systems should always provide PGHD to stroke survivors to keep them informed about what is going on with their health status, through appropriate feedback within reasonable time. This would increase their understanding about their rehabilitation or therapy process.
3	PGHD-enabled systems should avoid formatting PGHD through a scale that represents a completeness or an endpoint, for example, 100% as much as possible, as it would likely represent failure. Instead, PGHD should resemble the ongoing functional therapeutic progress of stroke survivors. Matched with original heuristic 2.	PGHD-enabled systems should present PGHD in a format that matches the real-world context, therapy progress, and goals of the stroke survivors.
4	PGHD-enabled systems should ensure that PGHD is, or could be, presented in a way that is clearly understandable to a stroke survivor. Matched with original heuristic 2.	PGHD-enabled systems should ensure that PGHD is clearly understandable to stroke survivors. The system should speak their language, with words, phrases, and concepts familiar to them, rather than system-oriented terms. PGHD-enabled systems should follow real-world conventions, making information appear in a natural and logical order.
5	PGHD-enabled systems should provide PGHD to stroke survivors for increased self-awareness about their health care. Matched with original heuristic 1.	PGHD-enabled systems should always provide PGHD to stroke survivors to keep them informed about what is going on with their health status, through appropriate feedback within reasonable time. This would increase their self-awareness about their health care.
6	PGHD-enabled systems should provide patients real-time PGHD that allow them to self-manage their energy while performing therapy exercises. Matched with original heuristic 1.	PGHD-enabled systems should always provide PGHD to stroke survivors to keep them informed about what is going on with their health status, through appropriate feedback within reasonable time. This would allow them to self-manage their energy while performing therapy exercises.
7	Notwithstanding the need for PGHD to be as accurate as possible, PGHD-enabled systems should inform the patients of its limitations or potential inaccuracies in the PGHD produced by stroke survivors. PGHD-enabled systems should also provide PGHD to stroke survivors to foster an increased sense of trust about their rehabilitation or therapy process. Matched with original heuristic 1.	PGHD-enabled systems should always provide PGHD to stroke survivors to keep them informed about what is going on with their health status, through appropriate feedback within reasonable time. This would foster an increased sense of trust about their rehabilitation or therapy process.
8	PGHD-enabled systems should allow stroke survivors to contact their/a clinician about their PGHD or at least provide survivors with the option of viewing functional, action-based suggestions for them to improve their performance. Matched with original heuristic 10.	Even though it is better if the system can be used without additional help or documentation, it may be necessary to provide them. PGHD-enabled systems should provide stroke survivors the option to contact a clinician about their PGHD and vice versa or at least provide survivors with the option of viewing functional, action-based suggestions for them to improve their performance. Any such information should be easy to search, focused on the survivors’ exercises, list concrete steps to be carried out, and not be too lengthy.
9	PGHD-enabled systems should provide stroke survivors the option of allowing their clinicians to contact them based on the progress of their PGHD. Matched with original heuristic 10.	Even though it is better if the system can be used without additional help or documentation, it may be necessary to provide them. PGHD-enabled systems should provide stroke survivors the option to contact a clinician about their PGHD and vice versa or at least provide survivors with the option of viewing functional, action-based suggestions for them to improve their performance. Any such information should be easy to search, focused on the survivors’ exercises, list concrete steps to be carried out, and not be too lengthy.

^a^PGHD: person-generated health data.

**Characteristics defined from key concepts identified from steps 1 and 2, which do not have a matching original heuristic.**

**Characteristics**

- 10
  Person-generated health data (PGHD)–enabled systems should present PGHD that indicate negative or decreasing therapy progress carefully and in a form that elicits a stroke survivors’ competitiveness with the self.
- 11
  PGHD-enabled systems should highlight PGHD indicating positive or improving therapy progress more and providing them with more frequency than negative or decreasing progress.
- 12
  PGHD-enabled systems should provide PGHD to stroke survivors for increased understanding about how their rehabilitation is contributing to their functional ability.
- 13
  PGHD-enabled systems should provide stroke survivors with the option to share their PGHD with loved ones in a secure manner.

### Step 4: Specification Stage

In this stage, the characteristics defined in step 3 are specified as a heuristic, following a structured format: ID, name, definition, explanation of how the heuristic was developed, example(s) of when a system being evaluated complies with or violates the heuristic, expected benefits if the system complies with the heuristic, and anticipated problems of heuristic misunderstanding [45].

The ID applied to the heuristics was structured as [Number]-PGHD-W[Weight]. The weights were indicated to provide implementers an idea of the process that went through developing the heuristics. The heuristic names were written succinctly, in a similar fashion to the original heuristics [29]. The characteristics were written as the heuristic definition. The explanation of each heuristic described how they were developed from one or more heuristic and provided example descriptions of those concepts by stroke survivors, clinicians, or both. The example and benefits of each heuristic were described based on the literature review [14] and qualitative studies [1]. The anticipated problems indicated where heuristic definitions may be quite close and may be misunderstood.

A number of characteristics were either similar or tightly complemented each other and were therefore combined into one heuristic. Characteristics 3 and 12 described the need for K-SRS to match the health and therapy context of the stroke survivors. Characteristics 5, 2, 6, and 7 described the need for K-SRS to always provide PGHD to stroke survivors. Finally, characteristics 8 and 9 both described the need for K-SRS to provide options for stroke survivors to seek more information, contact their clinicians, and allow their clinician to contact them.

A necessary augmentation is conducted in this step. In combining the characteristics, considerations had to be taken with the individual weights and the categories of those characteristics. Where the weights of the characteristics being combined were different, the highest weight was indicated for the resulting heuristic’s ID. Meanwhile, the categories were added as tag(s) under each heuristic and reworded to indicate how the heuristic informs implementers to enable PGHD use. This preserves the categories identified in step 2 as metadata that could aid implementers in understanding the concepts underlying each heuristic. Interestingly, the R3C methodology did not describe how the categories were relevant to the resulting heuristics [41].

The heuristics were initially specified by GD and then independently reviewed by the coauthors. Any disagreements were discussed in a meeting until agreement was reached for each specified heuristic. Moreover, steps 5 and 6 are expected to refine the example, expected benefits, and anticipated problems of each heuristic. Therefore, the heuristics presented here may still be revised later on [45].

The next section shows the new, initial set of heuristics for PGHD enablement.

### Design Heuristics to Enable Better Use of PGHD for Testing

Eight new heuristics for PGHD enablement in K-SRS were created. Heuristics with higher weights are presented first, to highlight heuristics that should be prioritized. The first 6 heuristics have a weight of 3, and the last 2 heuristics have a weight of 2. There are 6 tags used: improve health-related behaviors, increase positive feelings about health status, facilitate positive personal care goals, increase interest in care processes, improve relationships with care providers, and improve relationships with family and caregivers.

For brevity, only the ID, name, tag(s), definition, and explanation are presented in Textbox 3. This could also be the short form that implementers may use when evaluating HITs, similar to how brief definitions are presented for the original heuristics [29]. However, as with the original heuristics, implementers ought to understand the underlying concepts and anticipated problems of each heuristic and should, therefore, view the full specifications in Multimedia Appendix 1.

**Short form of the specified heuristics for person-generated health data enablement.**

1-PGHD-W3: Encouraging person-generated health data (PGHD)

- Tag(s): Improve health-related behaviors; increase positive feelings about health status
- Definition: The system should highlight PGHD indicating positive or improving therapy progress and provide them with more frequency than negative or decreasing progress.
- Explanation: This heuristic was formed from 3 concepts identified through a literature review and confirmed by stroke survivors and clinicians. Stroke survivors and clinicians commented on how PGHD can cause positive or negative emotions, correlated with whether the PGHD is positive or negative, for example, when survivors frequently see their PGHD improve, it encourages them to do more of the exercises. A clinician described how seeing PGHD could help survivors be more compliant. On the other hand, if survivors see their PGHD decline, they might lose faith in the system and not perform their exercises.

2-PGHD-W3: Evoking competitiveness with self

- Tag(s): Facilitate positive personal care goals
- Definition: The system should present PGHD that indicates negative or decreasing therapy progress carefully and in a form that elicits a stroke survivors’ competitiveness with the self.
- Explanation: This was a concept identified through a literature review and confirmed by stroke survivors and clinicians. Stroke survivors described how seeing their PGHD can motivate them to try harder, sometimes even when their PGHD indicates that they are not doing very well with their therapy exercises, because it can feel like a competition with themselves. Clinicians agree that it can give survivors something to keep them striving to be better.

3-PGHD-W3: Understandable health data

- Tag(s): Increase positive feelings about health status
- Definition: PGHD-enabled systems should ensure that PGHD is clearly understandable to stroke survivors. The system should speak their language, with words, phrases, and concepts familiar to them, rather than system-oriented terms. They should follow real-world conventions, making information appear in a natural and logical order.
- Explanation: This was a concept identified through a literature review and confirmed by stroke survivors and clinicians. A stroke survivor found that simply having percentages as feedback for performing an exercise could be confusing, as it could be misunderstood as being compared with people who have not had stroke. Clinicians also agreed that PGHD may not be understood by survivors.

4-PGHD-W3: Visibility of health progress

- Tags: Increase positive feelings about health status; increase interest in care processes
- Definition: PGHD-enabled systems should always provide PGHD to stroke survivors to keep them informed about what is going on with their health status, through appropriate feedback within reasonable time.
- Explanation: This heuristic was formed from 4 concepts identified through a literature review and confirmed by stroke survivors and clinicians. Stroke survivors described how PGHD could help them understand their rehabilitation progress, to determine whether their functional progress was headed in the right direction and learn when they have made a mistake. PGHD could also help them self-manage their energy as they perform their exercises and increase their trust in their therapy process.

5-PGHD-W3: Help and support

- Tag(s): Improve relationship with care providers
- Definition: Even though it is better if the system can be used without additional help or documentation, it may be necessary to provide them. PGHD-enabled systems should provide stroke survivors the option to contact a clinician about their PGHD; allow their clinicians to contact them; or at least provide survivors with the option of viewing functional, action-based suggestions for them to achieve their therapy goals. Any such information should be easy to search, focused on the survivors’ exercises, list concrete steps to be carried out, and not be too lengthy.
- Explanation: This heuristic was formed from 2 concepts identified through a literature review and confirmed by stroke survivors and clinicians. Stroke survivors described how they would want to contact their therapists, particularly if they have not been doing well to ask for assistance and ask for advice on what they could do better. Clinicians also described how it would be good if therapists could contact survivors when they are not being compliant. They also described how stroke survivors would likely ask for more explanation and for advice on how they can do things differently to improve their PGHD.

6-PGHD-W3: Communication of health data

- Tag(s): Improve relationship with family and carers
- Definition: PGHD-enabled systems should provide stroke survivors with the option to share their PGHD with concerned parties, for example, loved ones, in a secure manner.
- Explanation: This was a concept identified through a literature review and confirmed by stroke survivors and clinicians. Stroke survivors described how it could help them communicate their health status to people they would like to share it with, particularly when they are tired or when they “run out of words.” Clinicians described how survivors might want to share their PGHD with family, particularly with the younger generation who are more technologically inclined.

7-PGHD-W2: Guide for correct exercise movement or actions

- Tag(s): Improve health-related behaviors
- Definition: The system should help stroke survivors in performing more exercises or actions and to recognize, understand, and recover from errors they make. Guidance or error messages should be expressed in plain language (no codes), precisely indicate the problem, and constructively suggest a solution.
- Explanation: This was a concept identified through a literature review and confirmed by stroke survivors. A stroke survivor commented on how PGHD can help them work out what they were doing wrong and adjust accordingly.

8-PGHD-W2: Match between system PGHD and real-world context of stroke survivors

- Tag(s): Facilitate positive personal care goals; increase interest in care processes
- Definition: PGHD-enabled systems should present PGHD in a format that matches the real-world context, therapy progress, and goals of the stroke survivors. When presenting PGHD, for example, as a score, percentage, or a graph, they should be matched to the therapy goals of the survivors. These goals may change over the course of a survivors' therapy, from gaining functional progress in the first few months or years to maintaining function to perform ADL when their progress starts to plateau [1]. PGHD needs to be connected with the needs of the stroke survivors and where they are clinically.
- Explanation: This heuristic was formed from 2 concepts identified through a literature review and confirmed by stroke survivors and clinicians. Stroke survivors described how receiving a percent score that did not indicate a 100% could be a negative motivator. Achieving 100% given their condition could be very difficult or even impossible. Clinicians also described how PGHD should help survivors understand how their therapy is contributing to improved performance of ADL.

## Discussion

### Principal Findings

This study is the first to develop a new set of specialized heuristics to evaluate how HITs incorporate features that enable effective patient use of PGHD, with K-SRS as a key case study. In addition, it is the first to describe how the identification of initial HIT features and concepts to enable PGHD could lead to the development of a specialized set of heuristics. As such, it uncovers a valuable dimension to the PROM-PGHD development method, which considers the sociotechnical context of HITs [15,22]. This context is the complex nature of interactions between people’s unique health conditions and behavior and that of the technologies and tools they use within their environment [53]. The prior development of a PROM-PGHD for the key case of K-SRS was guided by this context [15]. Although PROMs have long been known to support the evaluation of health interventions, which may be supported by HITs [20], they are developed to consider only the health condition of patients. Conversely, the nature of PGHD requires the development of a PROM-PGHD to consider the sociotechnical context of the patient experience, that is, both the health condition and technology category [15].

Subsequently, this study highlights the value of considering the sociotechnical context of HITs in their design and evaluation. Although unexpected consequences and even failures can occur from design flaws and technical limitations, they are also often the result of sociotechnical factors [54]. Thus, HIT usability testing and evaluation can benefit from frameworks, standards, and guidelines that consider the sociotechnical context of those technologies [53,55]. In particular, PROMs-PGHD [15], heuristics for PGHD enablement, and cognitive work analysis [56] ensure that understanding of the complexities of care is incorporated in HIT design and implementation [56].

The R3C methodology [45] guided the heuristic development process; however, it was not applied without some difficulty. The methodology’s development, description, and application to a case study [44,47] were not described in explicit detail, which left too much room for flexibility. More specifically, it did not specify how the concepts identified from step 1 were going to be connected in step 2; how weights were going to be determined and applied to those concepts, and what influence those weights would have on the succeeding steps; how the characteristics were going to be identified in step 3 and how they differed from the concepts previously identified; and how the original heuristics were going to be “used” as a basis, after matching them with the defined characteristics. Moreover, although R3C presented a structured template for the specification of the heuristics in step 4, the resulting case heuristics only presented summaries or abbreviated versions [47,57,58]. Subsequently, implementations of R3C’s steps to develop other specialized heuristics have varied [59-61].

A step-by-step implementation of the R3C methodology was presented. A systematic and detailed process was applied in each step of heuristic development, which bridged the gaps described earlier. It is hoped that this would aid future developers of specialized heuristics, who could apply the detailed process of heuristic development for other domains of technology, and additionally for the case of PGHD enablement for other health conditions. Throughout the process, it was observed that the concepts and characteristics may need to be combined. This study presents a way to retain the weights and categories applied to them as heuristic metadata to aid implementers in understanding their underlying concepts. Although the last 2 heuristics have a weight of 2, implementers should still aim to conduct the evaluation using all 8 heuristics. However, in case of any resource constraint, the first 6 heuristics should be prioritized.

The R3C methodology [44] was also augmented through the use of qualitative studies with target users (stroke survivors) and domain experts (physiotherapists) to determine the weights, discover new concepts, and ensure the defined characteristics and resulting heuristics were as close to the experiences of the users as possible. This is intended to result in a robust and credible set of heuristics, before the validation stage (step 5), after which the specified heuristics may be improved through the refinement stage (step 6). This is especially valuable, as the R3C methodology’s recommended iteration of the process leads from step 6 back to step 4 [45], implying that steps 1 to 3 need to be rigorously implemented. Subsequently, the initial set of heuristics presented here will be validated and refined following steps 5 and 6.

### Limitations

Data gathered from the qualitative studies were used as an important augmentation in the heuristic development process. However, as those studies were primarily meant to support the development of a PROM-PGHD, it is possible that participants were not asked all the relevant questions around PGHD enablement features. In the event, many concepts found through the literature review were supported by qualitative studies, and indeed, most of the resulting heuristics were formed from new concepts found in those studies. Therefore, although it might appear to be a limiting factor, it actually indicates the robustness of the process followed and highlights the importance of this suggested augmentation. Moreover, the validation stage (step 5) still allows for possible additional heuristics to be identified.

The flexibility of the R3C methodology [44] meant that it is possible that the process followed in this study deviated from the original intent of the authors. However, we believe that the detailed, step-by-step implementation of R3C and the suggested augmentation have in effect modeled a way to build on and strengthen it.

### Conclusions

The new set of heuristics for PGHD enablement, following a detailed, systematic development process augmented from best practice that we have presented, could serve as a guide for future developers of specialized heuristics in general and specifically for developers of heuristics for PGHD enablement of a variety of technology domains and health conditions. The new set of heuristics is needed in a period of rising demand for, supply of, and variety of PGHD-enabled technologies in health care. It offers health care providers a standard way to evaluate technologies as part of selecting and implementing PGHD programs with their patients [11], complementing other in-depth PGHD evaluation methods [2,15], and has a broader relevance for the design and implementation of HITs. In addition, an interesting dimension to the PROM-PGHD development process was discovered, and it highlights the value of considering the sociotechnical context of HITs in their design and evaluation.

## Appendix

Multimedia Appendix 1 Steps 1-4, transforming concepts to heuristics.

## Abbreviations

ADL: activities of daily living
HE: heuristic evaluation
HIT: health information technology
K-SRS: Kinect-based stroke rehabilitation system
PGHD: person-generated health data
PROM: patient-reported outcome measure
PROM-PGHD: patient-reported outcome measure of utilizing person-generated health data
